# Key Factors Influencing Rates of Heterotrophic Sulfate Reduction in Active Seafloor Hydrothermal Massive Sulfide Deposits

**DOI:** 10.3389/fmicb.2015.01449

**Published:** 2015-12-22

**Authors:** Kiana L. Frank, Karyn L. Rogers, Daniel R. Rogers, David T. Johnston, Peter R. Girguis

**Affiliations:** ^1^Department of Molecular Biology, Harvard UniversityCambridge, MA, USA; ^2^Department of Oceanography, University of HawaiiHonolulu, HI, USA; ^3^Department of Earth and Environmental Sciences, Rensselaer Polytechnic InstituteTroy, NY, USA; ^4^Department of Chemistry, Stonehill CollegeEaston, MA, USA; ^5^Department of Earth and Planetary Sciences, Harvard UniversityCambridge, MA, USA; ^6^Department of Organismic and Evolutionary Biology, Harvard UniversityCambridge, MA, USA

**Keywords:** sulfate reduction, rate, energetics, hydrothermal deposits, microbial activity

## Abstract

Hydrothermal vents are thermally and geochemically dynamic habitats, and the organisms therein are subject to steep gradients in temperature and chemistry. To date, the influence of these environmental dynamics on microbial sulfate reduction has not been well constrained. Here, via multivariate experiments, we evaluate the effects of key environmental variables (temperature, pH, H_2_S, ${\text{SO}}_{4}^{2-}$, DOC) on sulfate reduction rates and metabolic energy yields in material recovered from a hydrothermal flange from the Grotto edifice in the Main Endeavor Field, Juan de Fuca Ridge. Sulfate reduction was measured in batch reactions across a range of physico-chemical conditions. Temperature and pH were the strongest stimuli, and maximum sulfate reduction rates were observed at 50°C and pH 6, suggesting that the *in situ* community of sulfate-reducing organisms in Grotto flanges may be most active in a slightly acidic and moderate thermal/chemical regime. At pH 4, sulfate reduction rates increased with sulfide concentrations most likely due to the mitigation of metal toxicity. While substrate concentrations also influenced sulfate reduction rates, energy-rich conditions muted the effect of metabolic energetics on sulfate reduction rates. We posit that variability in sulfate reduction rates reflect the response of the active microbial consortia to environmental constraints on *in situ* microbial physiology, toxicity, and the type and extent of energy limitation. These experiments help to constrain models of the spatial contribution of heterotrophic sulfate reduction within the complex gradients inherent to seafloor hydrothermal deposits.

## Introduction

Sulfate-reducing bacteria and archaea gain energy by mediating the anaerobic oxidation of organic and inorganic substrates using sulfate as an electron acceptor. The electron donors utilized by sulfate reducers are quite variable, ranging from molecular hydrogen to aromatic compounds, although low molecular weight organic compounds including acetate, lactate, pyruvate, and ethanol (prevalent in anaerobic environments as a result of the fermentative breakdown of biomass) are commonly used (Amend et al., 2004; Rabus et al., 2006; Tarpgaard et al., 2011).

Due to the high concentration of sulfate in seawater (28 mM; Dittmar, 1884), sulfate reduction (or SR) is an ubiquitous metabolism in hypoxic to anoxic marine habitats (Muyzer and Stams, 2008) such as oxygen minimum zone waters (Canfield et al., 2010), marine sediments (Kallmeyer et al., 2002; Robador et al., 2009), hydrocarbon seeps (Joye et al., 2004; Orcutt et al., 2005), marine hydrothermal vents (Jørgensen et al., 1992; Elsgaard et al., 1994a; Weber and Jørgensen, 2002; Frank et al., 2013), and the deep subsurface (D'Hondt et al., 2004; Engelen et al., 2008; Robador et al., 2015). As such, SR likely has a significant impact on the global carbon budget (Westrich and Berner, 1984; Canfield, 1989). Among 33 phylogenetically diverse sulfate-reducing microbial isolates, cell-specific SR rates vary by orders of magnitude in their respective thermal regimes (0.9 fmol cell^−1^ day^−1^ for *Desulfospira joergensenii* to 4340 fmol cell^−1^ day^−1^ for *Desulfohalobium retbaense*; Detmers et al., 2001). In marine sediments, estimates of cell-specific SR rates range from 10^−4^ to 10^0^ fmol cell^−1^ day^−1^ and are often lower than those of pure cultures by orders of magnitude (Jørgensen and Bak, 1991; Ravenschlag et al., 2000; Orcutt et al., 2005; Leloup et al., 2007, 2009; Holmkvist et al., 2011a,b). SR rates have also been shown to be highly heterogeneous within marine sediments due to variations in substrate and organic carbon availability, as well as other geochemical and physical factors (Canfield, 1989; Isaksen et al., 1994; Kallmeyer et al., 2002; Orcutt et al., 2005; Fike et al., 2008; Treude et al., 2009; Holmkvist et al., 2011a).

SR has been proposed to be one of the most thermodynamically favorable metabolic processes in marine hydrothermal ecosystems (McCollom and Shock, 1997; Tivey, 2004) because of the relative abundance of sulfate as an oxidant, as well as the relative abundance of appropriate reductants. Indeed, many sulfate-reducing microorganisms have been isolated from deep-sea hydrothermal ecosystems (Huber et al., 1997; Jeanthon et al., 2002; Alazard et al., 2003; Audiffrin et al., 2003). Data on the mass-specific SR rates in hydrothermal-influenced sediments (25–6600 nmol g-1 day-1, assuming an average sediment density of 2 g cm^−3^; Elsgaard et al., 1994a,b; Weber and Jørgensen, 2002; Kallmeyer and Boetius, 2004), sulfide deposits (16–2700 nmol g^−1^ day^−1^, Frank et al., 2013), and basaltic fluids (0.01–0.05 nmol ml^−1^ day^−1^, Robador et al., 2015) underscore the biogeochemical and ecological relevance of microbial sulfate reduction in deep-sea hydrothermal ecosystems.

At hydrothermal vents, structures called chimneys develop as hydrothermal fluid mixes with cold seawater, providing a unique if not ideal habitat for a variety of bacteria and archaea that rely on vent- and seawater-derived compounds (Schrenk et al., 2003). However, the microorganisms within these porous mineral deposits are exposed, in varying degrees, to the dynamic and combined effects of high temperature, low pH, high concentrations of hydrogen sulfide, fluctuating amounts of carbon, and varying energy sources. Previous studies reveal differences in prokaryotic populations among individual sulfide deposits (Harmsen et al., 1997; Takai et al., 2001; Schrenk et al., 2003; Kormas et al., 2006; Pagé et al., 2008), but we know less about how physico-chemical gradients influence the rates of most key microbial metabolisms, including SR, within the walls of actively venting hydrothermal chimney structures.

The metabolic energy available for SR is likely influenced by the specific composition of the end-member fluids and ambient seawater (Tivey, 1995, 2004; Zhu et al., 2007) as well as temperature, among other factors. Microbial SR rates in hydrothermal vent systems are governed by a combination of thermodynamic driving forces and the kinetics of catabolic reactions. In energy-limited systems, metabolic reaction rates tend to decrease proportionally with catabolic energy yields, and the thermodynamic factor in the complete rate equation has been modeled either as a function of the energetic costs of ATP synthesis (Jin and Bethke, 2005) or the energetic requirements of maintaining a membrane potential (LaRowe et al., 2012). Application of these models to SR rates in hydrothermal vent environments and marine sediments suggests that SR rates can be significantly impacted by thermodynamic driving forces when substrates (e.g., ${\text{SO}}_{4}^{2-}$, CH_4_, acetate) are limiting (Jin and Bethke, 2005; LaRowe et al., 2012). However, in environments with non-limiting sulfate or DOC concentrations, such as the experiments described here, heterotrophic SR rates can be modeled with standard Michaelis-Menten kinetics (Jin and Bethke, 2005). Even then, kinetic models depend on metabolism- and species-specific model parameters (Jin and Bethke, 2005; LaRowe et al., 2012). Model-predicted rates of hydrogenotrophic SR in hydrothermal chimneys (800 pmol cm^3^ day^−1^; LaRowe et al., 2014) are two to four orders of magnitude less than heterotrophic SR rates reported from Middle Valley chimneys (Frank et al., 2013), suggesting that kinetic models do not adequately capture the complexities of the natural environment. Although both thermodynamics and kinetics are functions of the physico-geochemical environment, the extent to which environmental factors—and their dynamics—influence SR rates remains unknown in hydrothermal chimneys and must be experimentally determined.

To better understand the parameters that affect SR rates within actively venting hydrothermal chimneys, we conducted a series of experiments that examined rates of microbial heterotrophic SR in batch incubations over a range of environmentally relevant chemical conditions (pH, H_2_S, ${\text{SO}}_{4}^{2-}$, and organic carbon) and temperatures (4, 50, and 90°C) from hydrothermal flange recovered from the Grotto edifice at the Main Endeavor Field, Juan de Fuca Ridge. This vent was specifically selected to be the focus of this study because its geology, mineralogy (pyrrhotite and pyrite), and end-member fluid chemistry (pH of 4.2, 12.6 mmol kg^−1^ CO_2_, 633 μmol kg^−1^ NH_3_, 5.4 mmol kg^−1^ H_2_S, 11 μM DOC) have been extensively studied over the past decade (Tivey and Delaney, 1986; Delaney et al., 1992; Butterfield et al., 1994; Tivey et al., 1999, 2002; Tivey, 2004; Lang et al., 2006; Zhu et al., 2007). While models infer that sulfate concentration may govern SR within sulfide deposits, the discrepancies between modeled and empirically derived rates lead us to conclude that other factors may be responsible for the observed SR rates. We therefore posit that SR rates in sulfide deposits are controlled by other factors, such as hydrogen sulfide concentration, pH, or organic carbon availability. Here we present empirical evidence for SR activity within active hydrothermal chimneys and quantitatively constrain the magnitude of net heterotrophic SR activity across a gradient of environmentally-relevant physico-chemical regimes.

## Materials and methods

### Geologic setting and sampling of hydrothermal sulfide deposits

The Main Endeavor vent field is located at 47°57′ N and 129°06 W (Tivey and Delaney, 1986) at a depth of 2220 m, the most shallow portion of the Endeavor segment of the Juan de Fuca ridge. This vent field is comparable in size and quantity of hydrothermal chimneys to most other hydrothermal areas, and is characterized by many (>15) large steep-walled active sulfide deposits (containing multiple high temperature spires and diffusing flanges) surrounded by a scattering of smaller inactive hydrothermal sulfide deposits in a roughly 400 m^2^ area (Delaney et al., 1992; Robigou et al., 1993).

A single massive piece of hydrothermal deposit (~100 kg in weight) was recovered from a flange on the Grotto vent (47.949, −129.098) at a depth of 2188.3 m (Dive J2-575, AT-18-08, *R/V Atlantis*) and brought up to the surface in the basket of the *ROV Jason II*. This sample will be hereafter referred to as a “flange.” Once on board ship, tubeworms and other macrofauna were removed from the samples and the large pieces were broken into more manageable fragments (~10–20 cm^3^) with a flame-sterilized chisel and sledgehammer, with the user wearing sterile nitrile gloves. Samples were quickly transferred to 0.2 μm-filtered anaerobic (nitrogen-sparged) seawater. Samples were further broken down into smaller sizes while in anaerobic water, and subsamples from the interior of the fragments were immediately transferred to gastight jars (Freund Container Inc.) filled with sterile anaerobic seawater containing 2 mM sodium sulfide at pH 6, and stored at 4°C for incubations and analyses. The sterile sulfidic seawater in the gastight jars were refreshed periodically during storage at 4°C. The majority of the rate experiments (80%) were set up immediately on the ship using freshly collected samples. In parallel, subsamples (~1 cm^3^) from each flange were preserved aboard ship in glutaraldehyde (2.5% in phosphate buffered saline, PBS, pH 7.0), then prepared for electron microscopy via ethanol dehydration and critical point drying before being sputtered with a thin layer of gold-palladium to improve image resolution. Samples were imaged with a Zeiss model EVO Scanning Electron Microscope (SEM).

### Measuring sulfate reduction rates

#### Experimental design and incubation

Prior to incubation, each flange subsample was pulverized by hand for about 1 h to minimize fine-scale geological and microbial heterogeneity and facilitate more accurate experimental replication (akin to slurry experiments in sediments; Fossing and Jørgensen, 1989; Jørgensen et al., 1992; Weber and Jørgensen, 2002). Specifically, each subsample was pulverized with a flame-sterilized sledgehammer in sterile seawater actively bubbled with nitrogen within an anaerobic chamber. For each independent treatment, aliquots of 7.5 mL flange slurry (~29 g wet weight and 20 g dry weight) were transferred into Balch tubes in an anaerobic chamber, and supplemented with 15 mL of sterile artificial seawater media designed to mimic the geochemical conditions within a hydrothermal flange (400 mM NaCl, 25 mM KCl, 30 mM CaCl_2_, 2.3 mM NaHCO_3_, 14 mM ${\text{NaSO}}_{4}^{2-}$, 1 mM H_2_S, and 50 μM dissolved organic carbon—consisting of equimolar proportions 10 μM of pyruvate, citrate, formate, acetate, lactate) under a pure nitrogen headspace.

Concentrations of sulfide, sulfate and dissolved organic carbon (DOC) were varied independently to investigate concentration dependent effects on the rates of SR. The range of experimental conditions tested was determined from previously published concentration profiles of aqueous species modeled as functions of temperature and position within the Grotto vent structure (Tivey, 2004). Concentrations were varied by orders of magnitude within the modeled ranges to simulate conditions representative of different mixing regimes between seawater and vent fluid (Table 1). The range of DOC (which we approximate as a mix of pyruvate, citrate, formate, acetate, lactate—most of which have been identified to varying degrees within vent fluid and are known carbon sources for heterotrophic SR in culture) concentrations tested were based on the average DOC concentrations measured within diffuse fluids at the Main Endeavor Field (Lang et al., 2006, 2010). Hydrogen sulfide was present as H_2_S (pK_a_ in seawater of 6.60) across all the conditions tested (Amend and Shock, 2001). Incubations were carried out at pH 4 (to simulate the pH of end-member Grotto vent fluid and the average calculated pH of mixed fluids in highly reduced zones within the flange; Tivey, 2004) as well as pH 6 (representative of the calculated pH in fluid mixing zones; Tivey, 2004). All the results are presented and discussed in the context of the initial measured media conditions. During incubation, it is plausible that the dissolution or precipitation of sulfide minerals (or other chemical reactions) may have affected the pH and chemical composition of the media. Due to the presence of the radioactive tracer, fluid pH could not be readily measured throughout the course of the experiment. Estimates of final fluid conditions calculated with EQ3/6 support the observation that changes in fluid composition are likely consistent across samples incubated under the same conditions.

**Table 1:** **Experimental media conditions**.

**Experiment**	**[H_2_S]**	**[Sulfate]**	**[DOC]^a^**	**pH^b^**	**Temperature °C**	**Innoculum^c^**
Δ [Sulfide]	0 mM	14 mM	50 μM	4, 6	4, 50, 90	Fresh
	10 μM	14 mM	50 μM	4, 6	4, 50, 90	Fresh
	100 μM	14 mM	50 μM	4, 6	4, 50, 90	Fresh
	1 mM	14 mM	50 μM	4, 6	4, 50, 90	Fresh
Δ [DOC]	1 mM	14 mM	0 μM	4, 6	4, 50, 90	Stored
	1 mM	14 mM	0.5 μM	4, 6	4, 50, 90	Stored
	1 mM	14 mM	5 μM	4, 6	4, 50, 90	Stored
	1 mM	14 mM	50 μM	4, 6	4, 50, 90	Stored
Δ [Sulfate]	1 mM	10 nM	50 μM	4, 6	50	Stored
	1 mM	1 μM	50 μM	4, 6	50	Stored
	1 mM	10 μM	50 μM	4, 6	50	Stored
	1 mM	100 μM	50 μM	4, 6	50	Stored
	1 mM	1 mM	50 μM	4, 6	50	Stored

*The base for each experiment was artificial seawater mix (KCl, CaCl2, NaCl) and 2.3 mM bicarbonate*.

a *Dissolved organic carbon amendments consisted of equimolar proportions of pyruvate, citrate, formate, acetate, and lactate*.

b *pH of media added to incubations*.

c *Homogenized hydrothermal flange used fresh (i.e., immediately after collection on the boat), or stored (used within 1 year of collection, storage in anaerobic sulfidic seawater at 4°C. SR rates measured 1 year after collection (stored) were not statistically different from sulfate reduction measurements made immediately after collection fresh)*.

Sufficient ^35^${\text{SO}}_{4}^{2-}$ was added to achieve 15 μCi of activity. Samples were incubated anaerobically for 1, 3, or 7 days at ambient seawater (4°C), thermophilic (50°C), and hyperthermophilic (90°C) temperatures. The range of temperatures considered was representative of different thermal regimes associated with the surface, outer layer and middle regions of hydrothermal chimneys (Schrenk et al., 2003; Tivey, 2004; Kormas et al., 2006). Negative controls consisted of samples amended with 28 mM molybdate to inhibit SR (Saleh et al., 1964; Newport and Nedwell, 1988). Three biological replicates were run for each treatment, and two biological replicates for each control.

Upon completion, reactions were quenched with the injection of 5 mL 25% zinc acetate, at pH 8 (i.e., 20-fold excess Zn), and all samples were frozen at −20°C for further analysis. Eighty percent of incubations were performed shipboard with freshly collected samples and the remaining 20% of incubations were completed within 1 year of collection. Select incubations performed on ship and replicated in the laboratory within 1 year of collection revealed no significant shift in observed SR rates due to the storage conditions (see **Figure 3**). However, even though the rates seemed unaffected, we have no data on the taxonomic composition of the sulfate reducing communities and cannot exclude the possibility that communities may have shifted during storage as has been shown to be the case for stored sediment cores (Lin et al., 2010).

#### Chromium distillation analysis

To determine SR rates, samples were thawed and the supernatant was removed and filtered through a 0.2 μm syringe filter. The homogenized flange that remained in the tube was washed three times with deionized water to remove any remaining sulfate. One gram (wet weight) of flange material was added to 10 mL of a 1:1 ethanol to water solution in the chromium distillation apparatus, and then degassed with nitrogen for 15 min to drive the environment anoxic. Hydrogen sulfide gas was evolved after the anaerobic addition of 8 mL of 12 N HCl and 10 mL of 1 M reduced chromium chloride, followed by 3 h of heating. The resulting hydrogen sulfide gas was carried via nitrogen gas through a condenser to remove HCl, and was then trapped as zinc sulfide in a 25% zinc acetate solution. While cold distillation methods for measuring SR using radiotracers have been developed and improved in recent years (Kallmeyer et al., 2004a; Røy et al., 2014), our experience has shown that—for metal-rich sulfide deposits—the hot chromium distillation method is sufficient in producing consistent results when run with the appropriate controls. To moderate potential artifacts of hot distillation methods including elevated rates in control samples, experiments were analyzed in triplicate, on different days and with different glassware to minimize cross-contamination, and any activity observed in “control” samples was deleted from the treatments. The radioactivity of the resulting sulfide (Zn^35^S) and the remaining sulfate from the supernatant (^35^${\text{SO}}_{4}^{2-}$) were measured via liquid scintillation counter in Ultima Gold scintillation cocktail (ThermoFisher Inc., Waltham, MA).

#### Calculating sulfate reduction rates

Rates were determined using the following calculation as in Fossing and Jørgensen (1989).

(1) $$\begin{array}{l} SRR=\frac{{\text{nSO}}_{4}^{2-}\cdot a\cdot 1.06}{\left(a+A\right)\cdot t} \end{array}$$

Where n${\text{SO}}_{4}^{2-}$ is the quantity (in moles) of sulfate added to each incubation (14 mM ^*^ 15 mL = 210 μmol), *a* is the activity (dpm) of the trapped sulfide, 1.06 is the fractionation factor between the hydrogen sulfide and sulfate pools (Jørgensen and Fenchel, 1974), *A* is the activity of the sulfate pool at the completion of the incubation and t is the incubation time (days). The rates are presented in units of nmol S g^−1^ day^−1^. As previously mentioned, SR rates are numerically presented as the difference in rates between experimental and the molybdate inhibited controls, further mitigating any potential artifacts caused by hot distillation methods.

#### Calculating *V_max_*, *K_m_*, and assumptions

The Michaelis-Menten kinetic parameters of maximum rate (V_*max*_) and half-saturation constant (*K*_*m*_) were determined using a linearization of SR rate (*V*) vs. sulfate concentration [*S*] data via the Hanes Woolf plot (Equation 2),

(2) $$\begin{array}{l} \frac{\left[S\right]}{V}=\frac{V_{max}\left[S\right]}{K_{m}+\left[S\right]} \end{array}$$

which is more accurate than the double reciprocal Lineweaver-Burk plot (Leskovac, 2003). The slope and X-intercept of the Hanes Woolf plot yield $\frac{1}{V_{max}}$ and *K*_*m*_ respectively. Sulfate concentrations plotted were initial concentrations as has been historically used when incubation conditions were short and sulfate concentrations were not depleted such that they significantly influenced SR rates (Pallud and Van Cappellen, 2006). Based on our previous study (Frank et al., 2013), we anticipated that the magnitude of SR rates would be on the order of nmol g^−1^ day^−1^. Given that the input sulfate concentrations range from μM to mM—three orders of magnitude greater than the rate of consumption—and changes in sulfate concentration could not be resolved with our methods, the maximum input of SR activity on the concentration of sulfate is 0.03%. Consequently, we use the initial concentration here without concern.

### Bioenergetic calculations

Potential energy yields of the different metabolisms available in the incubations depend on temperature and fluid compositions. To quantify the energy yield from heterotrophic sulfate reduction (Table 2) in each incubation values of overall Gibbs energy (Δ*G*_*r*_) were calculated according to:

(3) $$\begin{array}{l} \Delta G_{r}\;=\;\Delta G_{r}^{0}\;+\text{ RT lnQ} \end{array}$$

where $\Delta G_{r}^{0}$ is the standard Gibbs energy of reaction at *in situ* temperature and pressure conditions, R is the gas constant, T is the temperature (Kelvin), and Q is the activity product, defined as

(4) $$\begin{array}{l} Q\;=\;\Pi a_{i}^{vi} \end{array}$$

where *a*_*i*_ represents the activity of the *i*th species and *v*_*i*_is the stoichiometric reaction coefficient, which is positive for products and negative for reactants. Values of $\Delta G_{r}^{0}$ were calculated at 1 bar and incubation temperatures using the geochemical software package SUPCRT92 (Johnson et al., 1992) and additional thermodynamic data from Shock (1995). Activities of aqueous species were calculated using the geochemical speciation program EQ3 (Wolery, 1992a) based on the media composition described in Section Measuring Sulfate Reduction Rates and Table 1, with additional data from previously published work (Shock and Koretsky, 1993; Shock, 1995). For concentrations equal to zero, a value of 10^−13^ mol/kg was used as input. Resulting aqueous activities were used to calculate values of Δ*G*_*r*_ normalized for the number of electrons transferred in the redox for the reactions in Table 2. These reflect the metabolic energy available at the start of each incubation experiment for the complete oxidation of each organic acid, metabolisms that are documented among known sulfate reducers (Amend and Shock, 2001). Furthermore, to calculate the energy density in each incubation (as in Amend et al., 2011), it was assumed that the amended organic acids were the limiting reactant for all experiments when sulfate concentrations were in excess of 1 mM; otherwise sulfate was assumed to be limiting. While some sulfate reducers are known to produce carboxylic acid and alcohol intermediates, incomplete oxidation reactions were not considered here, as the goal of these calculations was to generate a broad understanding of sulfate reduction energetics, and not the metabolic potential for a particular species. Such an approach is common when comparing microbial metabolisms independent of species-specific pathways (e.g., Amend et al., 2004; Rogers and Amend, 2006; Skoog et al., 2007), although it should be noted that incomplete oxidation (fermentation) generally yields much less energy than complete oxidation (Rogers and Amend, 2006; Skoog et al., 2007).

**Table 2:** **Heterotrophic sulfate reduction**.

**Carbon source**	**Reaction**	***e^−^***
Formate	${\text{SO}}_{4}^{2-}$ + 4 CH_2_O_2_ 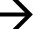 H_2_S + 4 ${\text{HCO}}_{3}^{-}$ + 2 H^+^	8
Acetate	${\text{SO}}_{4}^{2-}$ + C_2_H_4_O_2_ 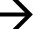 H_2_S + 2 ${\text{HCO}}_{3}^{-}$	8
Pyruvate	5 ${\text{SO}}_{4}^{2-}$ + 4 C_3_H_4_O_3_ + 4 H_2_O 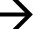 5 H_2_S + 12 ${\text{HCO}}_{3}^{-}$ + 2 H^+^	40
Lactate	3 ${\text{SO}}_{4}^{2-}$ + 2 C_3_H_6_O_3_ 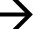 3 H_2_S + 6 ${\text{HCO}}_{3}^{-}$	24
Citrate	9 ${\text{SO}}_{4}^{2-}$ + 4 C_6_H_5_${\text{O}}_{7}^{3-}$ + 8 H_2_O + 6 H^+^ 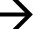 9 H_2_S + 24 ${\text{HCO}}_{3}^{-}$	72

To account for potential interactions between chimney-derived trace metals and amended sulfide, the saturation states of sulfide minerals were calculated as part of the initial fluid speciation (see above). Using reported concentrations of relevant trace metals (Fe, Zn, Cu, etc.) in end-member Grotto hydrothermal fluid (Butterfield et al., 1994), maximum aqueous activities of trace metals were calculated with the EQ3 geochemical speciation program (Wolery, 1992a,b). Several sulfide minerals commonly found in hydrothermal chimneys (e.g., pyrite, chalcocite, sphalerite) were supersaturated under incubation conditions, particularly for incubations with high concentrations of amended sulfide. The irreversible abiotic precipitation of mineral sulfides has the potential to draw down aqueous sulfide concentrations and impact sulfate reductions rates. Therefore, the geochemical reaction path program EQ6 (Wolery, 1992a; Wolery and Daveler, 1992) was used to constrain fluid compositions to equilibrium with these minerals phases. Using the single point model in EQ6, the Gibbs energy of the system was allowed to reach local minima by mineral precipitation, however redox reactions among carbon and sulfur species was suppressed with a custom thermodynamic database. The resulting fluid compositions were used to calculate metabolic reaction energetics as well as to evaluate the potential effects of metal speciation on sulfate reduction rates.

## Results

### Physical characteristics of the study site and microscopy

Diffuse hydrothermal flow was observed on the surface of the hydrothermal flange of Grotto ranging in temperature from 2 to 18.1°C, while hot hydrothermal fluid (*T*_*max*_ = 215.6°C) was observed in pools accumulating on the underside of an overhanging flange. Sulfide minerals nearest to the hottest fluid were relatively friable, whereas the majority of the flange sampled was relatively solid, likely due to the precipitation of pyrrhotite, pyrite, and silica (Tivey and Delaney, 1986) in pore spaces at lower temperatures. An inner conduit-like channel was clearly present in the interior of the flange and densely lined with pyrite. Thick veins of anhydrite and channels of marcasite were also observed throughout the samples. Crystals of these minerals were imaged using scanning electron microscopy. Microbial cells were observed associated with mineral surfaces in low abundance (Figure S1). There was no visual evidence of dense biofilms within the sample.

### Sulfate reduction rates

Sulfate reduction was detected in incubations of flange material recovered from the Grotto vent at 4, 50, and 90°C over a range of environmentally-relevant chemical conditions (pH, H_2_S, ${\text{SO}}_{4}^{2-}$, and organic carbon concentrations). Measured rates (except for those at low sulfate concentrations) are comparable in magnitude to those previously observed in hydrothermally-influenced sediments (e.g., Guaymas Basin or Lake Tanganyika; Elsgaard et al., 1994a,b; Weber and Jørgensen, 2002; Kallmeyer and Boetius, 2004). Among all treatments, the highest observed rate (3940 nmol g^−1^ day^−1^) occurred at 100 μM H_2_S, 14 mM ${\text{SO}}_{4}^{2-}$, 50 μM DOC, pH 6, and 50°C. The lowest observed rates (120 fmol g^−1^ day^−1^ – 1.5 nmol g^−1^ day^−1^) were under conditions of most pronounced sulfate limitation (10–100 μM) in the presence of 1 mM H_2_S, at pH 4 and 6, and at 50°C (it should be noted that we did not conduct these same sulfate limitation experiments at 4 and 90°C).

Control experiments were designed to inhibit biological sulfate reduction through the use of molybdate, a known inhibitor of SR. The SR rates in these controls were much lower than experimental samples (Mann-Whitney-Wilcox test, *p* = 0.1), though none of the samples were entirely inhibited, which is likely the result of mineral scavenging of molybdate (Bostick et al., 2003; Xu et al., 2006) or as an artifact of hot chromium distillation methods (Kallmeyer et al., 2004b). While we present rates for both experimental and molybdate-amended samples to provide a perspective on the relative contributions of abiotic vs. biotic sulfate reduction under varying environmental conditions (**Figures 2–4**), the subsequent analyses focus on a conservative measure of net biological rates, namely the difference in rates between experimental and the molybdate-inhibited controls. Additionally, because rates from 3-day to 7-day incubations were not significantly different from one another in these treatments (*n* = 271; Mann-Whitney-Wilcoxon, *p* = 0.728), they are being treated as pseudo-replicates. As has been previously observed (Frank et al., 2013), SR rates exhibited large standard deviations, presumably due to microscale sample heterogeneity (despite efforts to homogenize, mineral clasts ranged in size from 0.003 to 80 mm^3^) among biological replicates. Such variation is consistent with the patterns we observed in previous SR rate measurements on Middle Valley vent deposits (with mineral sizes 0.001–7 mm^3^; Frank et al., 2013).

**Figure 1:**
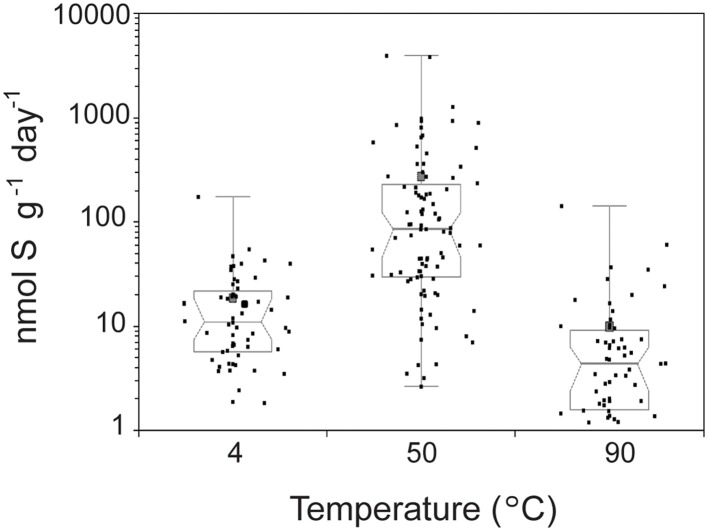
**Temperature dependence of sulfate reduction rates in crushed hydrothermal flange from Grotto vent (Main Endeavor, JdF)**. The median of 213 samples is plotted as a line, the 1st and 3rd quartiles as a box, and the minimum and maximum as whiskers with end caps. The 95% confidence interval of the median is represented by a notch on the box. The mean of the 213 samples is depicted as a gray square. Black squares indicate individual measurements. Note rates measured from sulfate gradient experiments were excluded in this figure because those experiments were only done at 50°C.

The highest SR rates across all conditions tested were observed at 50°C (Figure 1). The average SR rates with 14 mM sulfate were 16.4 nmol g^−1^ day^−1^ at 4°C (3.99–66.8 nmol g^−1^ day^−1^), 270 nmol g^−1^ day^−1^ at 50°C (10.0–1690 nmol g^−1^ day^−1^), and 7.66 nmol g^−1^ day^−1^ at 90°C (0–24.9 nmol g^−1^ day^−1^ at 90°C). SR rates observed at 50°C were significantly higher than those measured at 4°C (*n* = 152; Mann-Whitney-Wilcoxon, *p* < 0.0001) and 90°C (*n* = 151; Mann-Whitney-Wilcoxon, *p* < 0.0001). Notably, 60% of samples incubated at 90°C show no biological sulfate reduction. Our data show that when temperature is varied independently from chemistry (under thermodynamically favorable conditions of excess sulfate and no oxygen), the highest rates of SR occur at 50°C. Furthermore, the distribution of rates with respect to temperature resembles distributions observed in hydrothermal sediments (Jørgensen et al., 1992; Elsgaard et al., 1994a; Weber and Jørgensen, 2002) and basaltic crustal fluids (Robador et al., 2015), where optimal temperatures range between 40 and 80°C, but it differs from those measured in vent structures from Middle Valley, where maximal rates were observed at 90°C (Frank et al., 2013).

SR rates observed at pH 6 were significantly higher than those measured at pH 4 (*n* = 271; Mann-Whitney-Wilcoxon, *p* = 0.0267). The average SR rates at pH 6 were 149 nmol g^−1^ day^−1^ compared to 35.6 nmol g^−1^ day^−1^ at pH 4. Maximum SR rates at pH 6 seem incongruous with the pH of endmember hydrothermal fluid (4.2–4.7; Butterfield et al., 1994; Tivey, 2004), particularly in light of observations of Lake Tanganyika hydrothermal sediments, where maximum SR rates were observed at pH conditions near hydrothermal vent values (Elsgaard et al., 1994b). However, mixing models by Tivey (2004) predict fluid compositions with a pH of ~6 at 50°C when mixing is driven by seawater advection, suggesting that microbial communities adapted to the 50°C niche in the Grotto flange also have an optimal pH range close to estimated *in situ* values.

**Figure 2:**
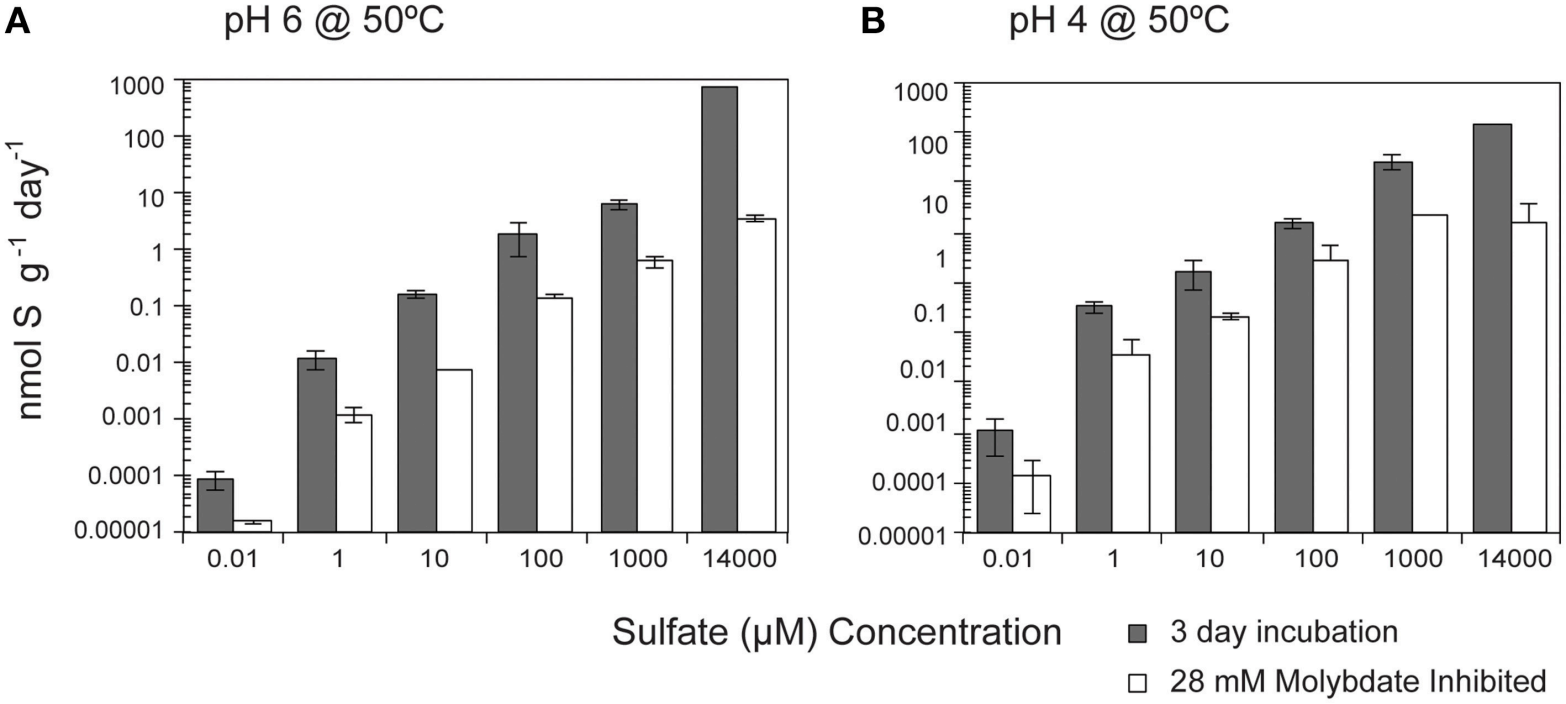
**Effect of sulfate concentration on sulfate reduction rates**. The crushed flange from the Grotto hydrothermal deposits were incubated for 3 days (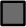) at 50°C at **(A)** pH 6 or **(B)** pH 4. 28 mM molybdate amended samples were incubated under all conditions (□) as controls. Error bars represent 1 standard deviation.

While temperature and pH were the dominant factors influencing SR rates, media amendments were also correlated with rates, though to a lesser degree. SR rates were positively correlated (*p* < 0.0001) with sulfate concentration (10–14 mM) at both pH 6 and pH 4 (Figure 2). Over the entire range of sulfate concentrations tested (seven orders of magnitude), SR rates ranged from 50.1 fmol g^−1^ day^−1^ to 930 nmol g^−1^ day^−1^ at pH 6 (seven orders of magnitude) and from 9.8 fmol g^−1^ day^−1^ to 805 nmol g^−1^ day^−1^ at pH 4 (eight orders of magnitude). Furthermore, SR rates were slightly higher at pH 4 when sulfate concentrations were less than 14 mM. Michaelis-Menten Kinetics parameters determined using the Hanes-Woolf linearized form of the Michaelis-Menten rate equation (Equation 2) revealed a *K*_*m*_ value of 7.1 mM and *V*_*max*_ of 298 nmol g^−1^ day^−1^ for SR at pH 6 (*r*^2^ = 0.441) and a *K*_*m*_ value of 2.5 mM and V_*max*_ of 298 nmol g^−1^ day^−1^ at pH 4 (*r*^2^ = 0.513). *K*_*m*_, defined as the sulfate concentration at which SR rates are half-maximum, is a measure of affinity for sulfate (higher *K*_*m*_ corresponds to lower affinity). Given that the *K*_*m*_ values for most enzymes in the sulfate reduction pathways range roughly from 5 to 100 μM in cultured organisms (Trüper and Rogers, 1971; Ingvorsen et al., 1981; Ingvorsen and Jørgensen, 1984; Sonne-hansen and Westermann, 1999; Ramos et al., 2012), the pattern of SR rates observed in these experiments suggests that sulfate reduction enzymes do not reach saturation at the amended sulfate concentrations (plausible explanations for this observation are presented in the discussion). The apparent *K*_*m*_ value for SR at pH 6 is higher than the highest reported *K*_*m*_ values for thermophilic SR (3.17 ± 1.02 mM) from Yellowstone Mushroom Springs (Roychoudhury, 2004). Interestingly, the apparent *K*_*m*_ values for SR at pH 6 are higher than at pH 4, even though *V*_*max*_ remains constant under both conditions. Due to the low *r*^2^ values for linear regressions, future experiments should consider a wider range of sulfate concentrations to validate these values.

**Figure 3:**
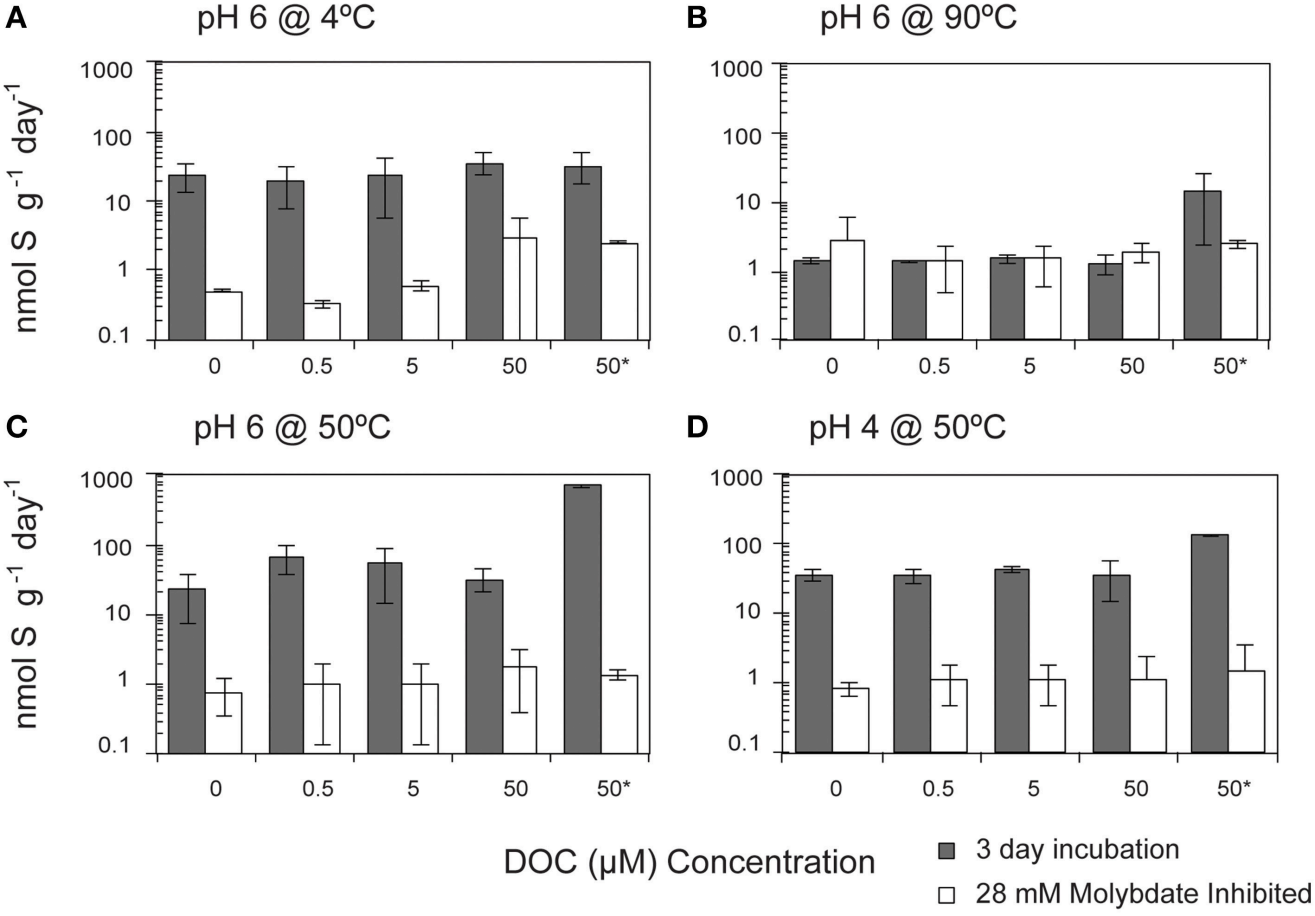
**Effect of dissolved organic carbon (DOC) concentrations on sulfate reduction rates**. The crushed flange from the Grotto hydrothermal deposits were incubated for 3 days (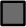) in media of **(A)** pH 6 at 4°C, **(B)** pH 6 at 90°C, **(C)** pH 6 at 50°C, and **(D)** pH 4 at 50°C. While most of experiments were performed in laboratory using stored samples, concentrations identified with an asterisk (^*^) were done shipboard immediately after collection of hydrothermal chimney. 28 mM molybdate amended samples were incubated under all conditions (□) as controls. Error bars represent 1 standard deviation. Note the log scale on the y-axis.

SR rates in the treatments without any exogenous DOC amendment were not significantly different from those incubated with DOC (pyruvate, citrate, formate, acetate, and lactate) at concentrations up to 50 μM (Pearson's correlation, *p* = 0.565; Figure 3). The observed rates suggest that the communities were not carbon limited and could have been utilizing organic carbon associated with flange materials. Given the common occurrence of macrofauna upon these chimneys, it is likely that endogenous organic carbon pools—such as from macrofaunal necromass—exist in these systems.

**Figure 4:**
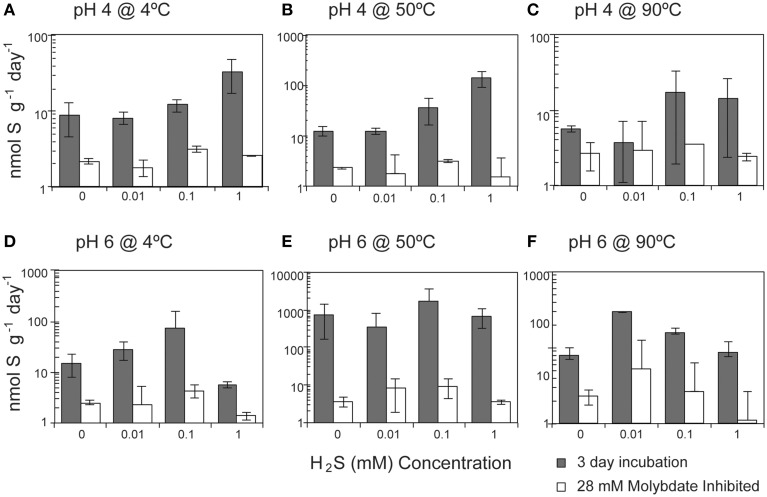
**Effect of sulfide concentration on sulfate reduction rates**. The crushed flange from the Grotto hydrothermal deposits were incubated for 3 days (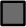) in media of **(A)** pH 4 at 4°C, **(B)** pH 4 at 50°C, **(C)** pH 4 at 90°C, **(D)** pH 6 at 4°C, **(E)** pH 6 at 50°C, and **(F)** pH 6 at 90°C. 28 mM molybdate amended samples were incubated under all conditions (□) as controls. Error bars represent 1 standard deviation. Note different scales for **(A,C)**.

Overall, SR rates (ranging from 1.9 to 3936 nmol g^−1^ day^−1^) were positively correlated with the concentration of hydrogen sulfide (Pearson's correlation, *n* = 213, *p* = 0.04; Figure 4). A strong correlation was observed at pH 4 at both 4°C (*p* < 0.0001) and 50°C (*p* < 0.0001), with SRR rates increasing between one and two orders of magnitude as the sulfide concentration increased from 0 to 1 mM (Table S1). In contrast, SR rates were not significantly correlated with the exogenous sulfide concentration at pH 6 (Pearson's correlation, *p* = 0.354).

### Bioenergetic models

**Figure 5:**
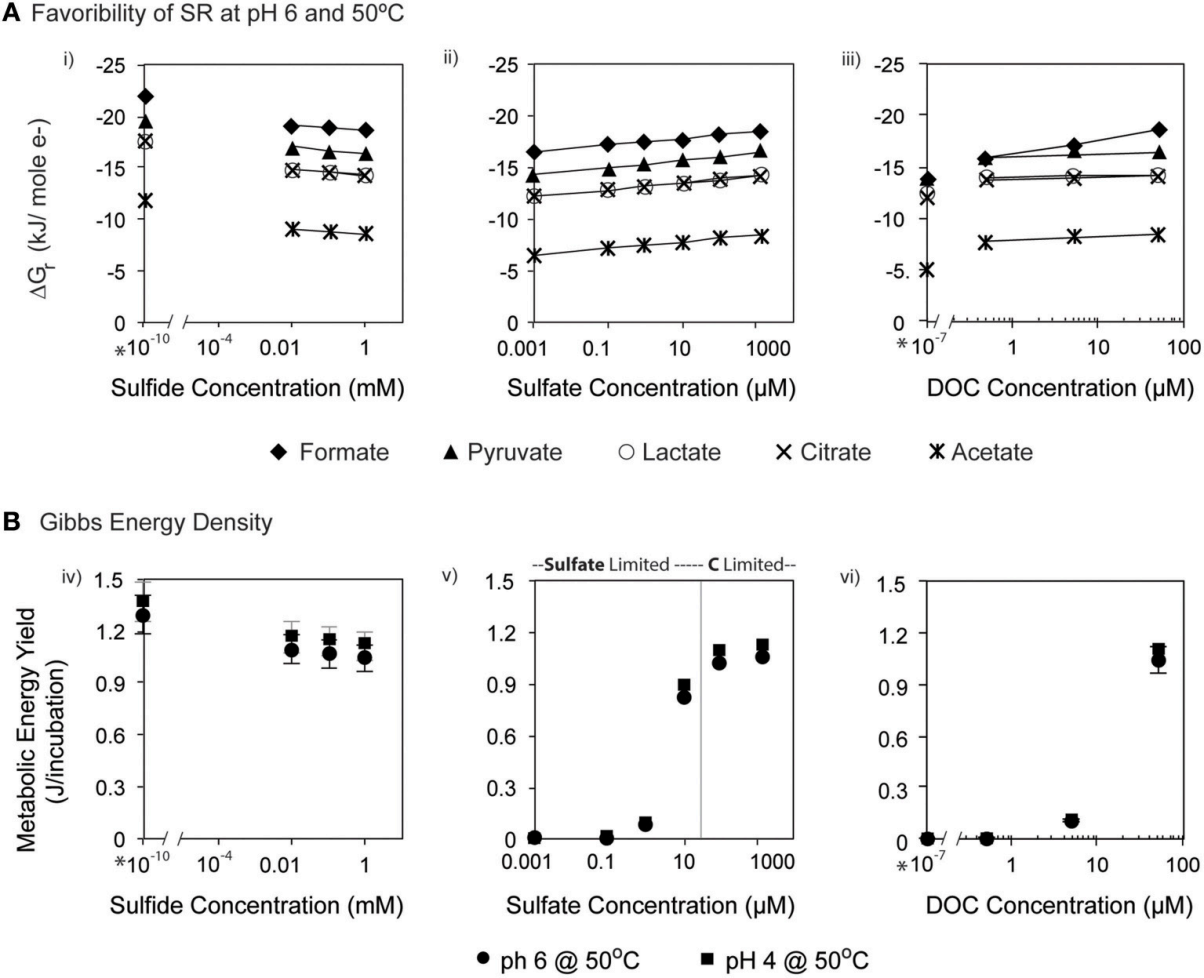
**(A)** Δ*G*_*r*_ (kJ/mol e^−^) calculated at pH 6 and 50°C for heterotrophic sulfate reduction via organic acids (formate, acetate, pyruvate, lactate and citrate) at varying concentrations of (i) sulfide, (ii) sulfate, and (iii) DOC. **(B)** The total energy available for sulfate reduction in each incubation at 50°C. Bars on each symbol represent the range of values for 4°C and 90°C. Asterisks (^*^) denote contrived values of zero that were inputted into the model for speciation calculation in EQ3.

The favorability of sulfate reduction in the incubation experiments, as estimated by overall Gibbs energy of reaction, Δ*G*_*r*_, ranged from −6.41 kJ/mol e^−^ to −20.7 kJ/mol e^−^ over the range of sulfate, DOC, and sulfide concentrations tested. Figure 5 shows Δ*G*_*r*_, normalized for the number of electrons transferred, for the range of conditions in the 50°C, pH 6 experiments. Note that negative values of Δ*G*_*r*_ indicate exergonic reactions, and higher negative values (increasing on the y-axis) reflect increasing energy levels. While sulfate, DOC, and sulfide concentrations were varied by five orders of magnitude or more, values of Δ*G*_*r*_ only changed by 2–3 kJ/mol e^−^ at this temperature and pH, emphasizing that *in situ* concentrations of individual electron donors, acceptors, or products are not the largest contributor to free energy variations. However, varying the type of electron donor (in this case the species of organic acid) effected changes in Δ*G*_*r*_ up to ~10 kJ/mol e^−^. For example, at 0.1 mM sulfate, oxidation of acetate yields −7.76 kJ/mol e^−^, while formate oxidation provides up to −17.79 kJ/mol e^−^. Not surprisingly, the highest calculated energy yields were from formate oxidation under the maximum sulfate and minimum sulfide concentrations (Figure 5Aiii). The effect of temperature on metabolic energy availability was less pronounced than the effect of the organic acid species, with only 1–2 kJ/mol e^−^ variations in Δ*G*_*r*_ for any given reaction evaluated at 4, 50, and 90°C (e.g., At pH 6, 0.1 mM H_2_S, 14 mM ${\text{SO}}_{4}^{2-}$, and 50 μM formate, Δ*G*_*r*_ ranged from −18.6 to −19.3 kJ/mol e^−^ from 4 to 90°C).

In batch experiments, like those described here, overall Gibbs energy values do not represent the amounts of substrate that might be limiting in closed systems. Accounting for the total amount of sulfate and DOC available for each incubation (by multiplying values of Δ*G*_*r*_ in kJ/mol rxn, by the concentration and stoichiometric reaction coefficient of the limiting substrate, as well as the volume of the incubation), it is possible to calculate the total amount of energy available (e.g., energy density; LaRowe and Amend, 2015) in each experiment (Figure 5B). Low concentrations of sulfate (${\text{SO}}_{4}^{2-}$-limited conditions) or DOC (organic carbon-limited conditions) both yielded minimal energy density (~10^−6^ kJ/incubation); however these values rose to nearly 1.2 kJ/incubation for concentrations of sulfate in excess of 1 mM and DOC at 50 μM (all under carbon limitation). Energy densities decreased minimally with increasing sulfide concentrations, consistent with expectations, as metabolic products are not limiting and therefore do not generally affect energy density.

While the species of electron donor, and—to a lesser extent—sulfate, sulfide, and individual organic acid concentrations, have some effect on overall metabolic energy yields, our calculations indicate that the largest contributor is the metabolic strategy itself. In general, Δ*G*_*r*_ values for both autotrophic and heterotrophic sulfate reduction occupy a small range of energetics over a broad range of environments compared to other metabolic strategies. For example, Rogers and Amend (2006) found that *in situ* Δ*G*_*r*_ values for SR coupled to oxidation of carboxylic acids ranged from −6.7 to −25.99 kJ/mol e^−^ in shallow marine hydrothermal vents, compared to −0.05 to −122.58 kJ/mol e^−^ for all other metabolic strategies considered (Amend et al., 2003; Rogers and Amend, 2006). Energy yields for heterotrophic SR from those shallow marine vents are very similar to values calculated here, despite having higher sulfate concentrations (up to 62 mM) and orders of magnitude lower sulfide (<400 μM) than the Grotto experiments. The metabolic energy yields calculated here are also similar to values predicted from seawater/hydrothermal fluid mixing models (McCollom and Shock, 1997; Rogers and Amend, 2006).

## Discussion

### Physiological constraints on SR rates

**Figure 6:**
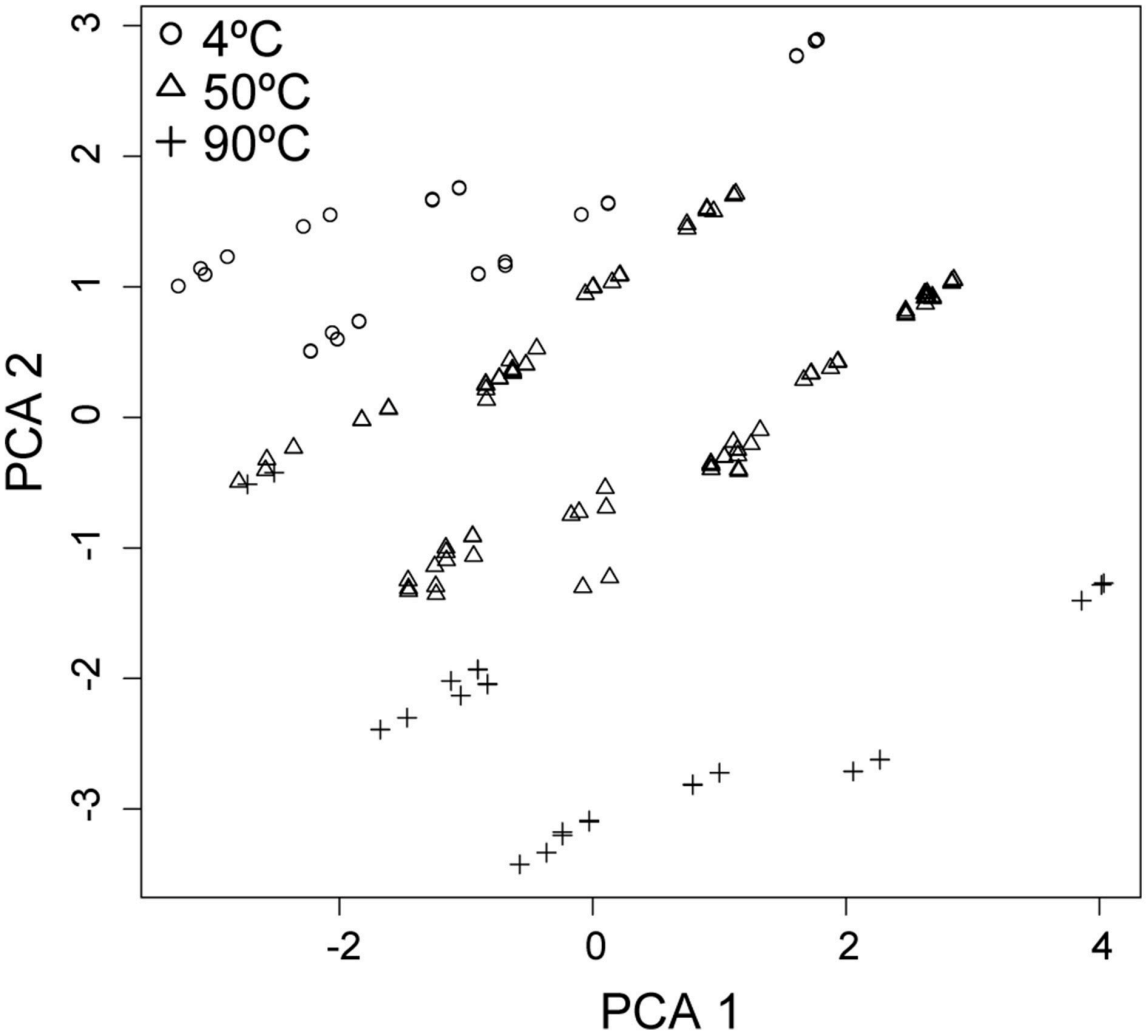
**Principal component analysis (PCA) of the variation between samples (***n*** = 274) as a linear combination of standardized chemical (sulfate, sulfide, pH, DOC, Cu^**2+**^, Fe^**2+**^, and Zn^**2+**^), thermal, rate and energetic parameters (variance within each parameter = 1)**. PCA 1 represents 28.5% and PCA 2 represents 19.9% of the variance within the data (PCA 3 and PCA 4, not shown, represent 12 and 10% respectively). Data are clustered most obviously by temperature at 4°C (○), 50°C (Δ), and 90°C (+).

The magnitude of SR rates within the Grotto flange is primarily governed by the temperature and, to a lesser extent, pH-dependent processes and their effect on the physiology of the native sulfate reducing microbial consortia (Figures 1, 2, 4, 6). As previously noted, the maximum rates of SR occurred at 50°C and pH 6, and suggest that the native SR community is most active in a moderate (~50°C) temperature and only slightly acidic pH regime. This is consistent with mixing models dominated by seawater advection, where a mixed fluid at 50°C had a pH of ~6, high sulfate, low sulfide and limited oxygen (note that these calculated concentrations of dissolved O_2_ do not take into consideration biotic or abiotic reactions that consume oxygen), providing ideal conditions for sulfate reduction in the outer region of the chimney (Tivey, 2004). The correspondence between our maximum SR rates and these mixing models suggests that advection of seawater into the wall of the vent may be an important factor for constraining the physiology of the native sulfate reducing community.

Previous work in other hydrothermal systems has also suggested that temperature is a primary factor in governing metabolic rates and, likely, community composition. For example, in basalt-hosted borehole fluids from Juan de Fuca Ridge maximum rates of SR occurred at temperatures near *in situ* conditions and were consistent with physiological tolerances of the native SR community (Robador et al., 2015). Similarly, in hydrothermal chimneys from Middle Valley vent field, Juan de Fuca Ridge, maximum SR rates were observed at temperatures (90°C) near the growth ranges for the *in situ* SR community (hyperthermophiles) (Frank et al., 2013). Considering that SR rates from Grotto and Middle Valley were measured under identical experimental conditions, differences in the thermal optima of SR between Grotto and Middle Valley are likely due to differences in the prokaryotic abundance and phylogenetic composition of associated consortia (Frank et al., 2013; Olins et al., 2013). This hypothesis is ecologically consistent with temperature being one of the main driving forces that shape the composition and abundance of hydrothermal vent microbial communities (Harmsen et al., 1997; Takai et al., 2001; Schrenk et al., 2003; Kormas et al., 2006; Pagé et al., 2008). Additionally, differences in density and composition within a chimney are a reflection of the mineralogy and physico-chemical conditions at each site. For example, deposits sampled at Middle Valley were physically smaller than Grotto, composed primarily of anhydrite (with no inner conduit like structures), and exposed to higher temperature end-member fluid (261°C). Thus, it is not surprising that the environment at Middle Valley selected for a hyperthermophilic community dominated by *Thermodesulfovibro* (Frank et al., 2013) or *Archaeoglobus*-like (Olins et al., 2013) organisms. On the other hand, Grotto is a very large hydrothermal deposit with a mature mineralogical structure (Tivey and Delaney, 1986), allowing for a greater range of habitable niches and more space for colonization. Because mesophillic, as well as slightly thermophilic phylotypes are often observed to colonize within the outer regions of hydrothermal vents (Takai et al., 2001; Hoek et al., 2003; Schrenk et al., 2003; Nakagawa et al., 2005; Kormas et al., 2006; Pagé et al., 2008), we posit that the majority of SR in the Grotto occurs in the outer to middle region of the flange and is dominated by thermophilic organisms.

### Environmental constraints on SR rates

Environmental selection of physiologically adapted microbial communities applies not only to parameters such as temperature and pH, but also to geochemical constraints such as dissolved sulfide and metal toxicity. Some SR isolates can be inhibited by elevated sulfide (2–15 mM) (Reis et al., 1992; Okabe et al., 1995; Koschorreck, 2008), although vent isolates are known to tolerate a wider range of sulfide concentrations. For example, archaea isolated from of deep-sea hydrothermal vents such as *Methanocaldococcus jannaschii, Archaeoglobus profundus, Thermococcus fumicolans*, and isolates from the genus *Pyrococcus* and *Desulfurococcus* have high tolerance to hydrogen sulfide concentrations in the range of 40–100 mM (Jannasch et al., 1988; Lloyd et al., 2005). Our incubations experiments were amended with sulfide at concentrations (up to 1 mM), well below the values measured in in Grotto end-member fluids (5.4 mmol/kg at pH = 4.2; Butterfield et al., 1994). Assuming that native Grotto SR communities are adapted to tolerate *in situ* sulfide concentrations, it is unlikely that sulfide toxicity largely impacted SR rates in our experiments. This is consistent with experiments at optimal temperature and pH (50°C, pH 6), for which SR rates do not significantly depend on sulfide concentrations (Table S1, *p* = 0.5), despite the fact that increasing sulfide concentrations do decrease energy yields (Figure 5Aiii).

**Figure 7:**
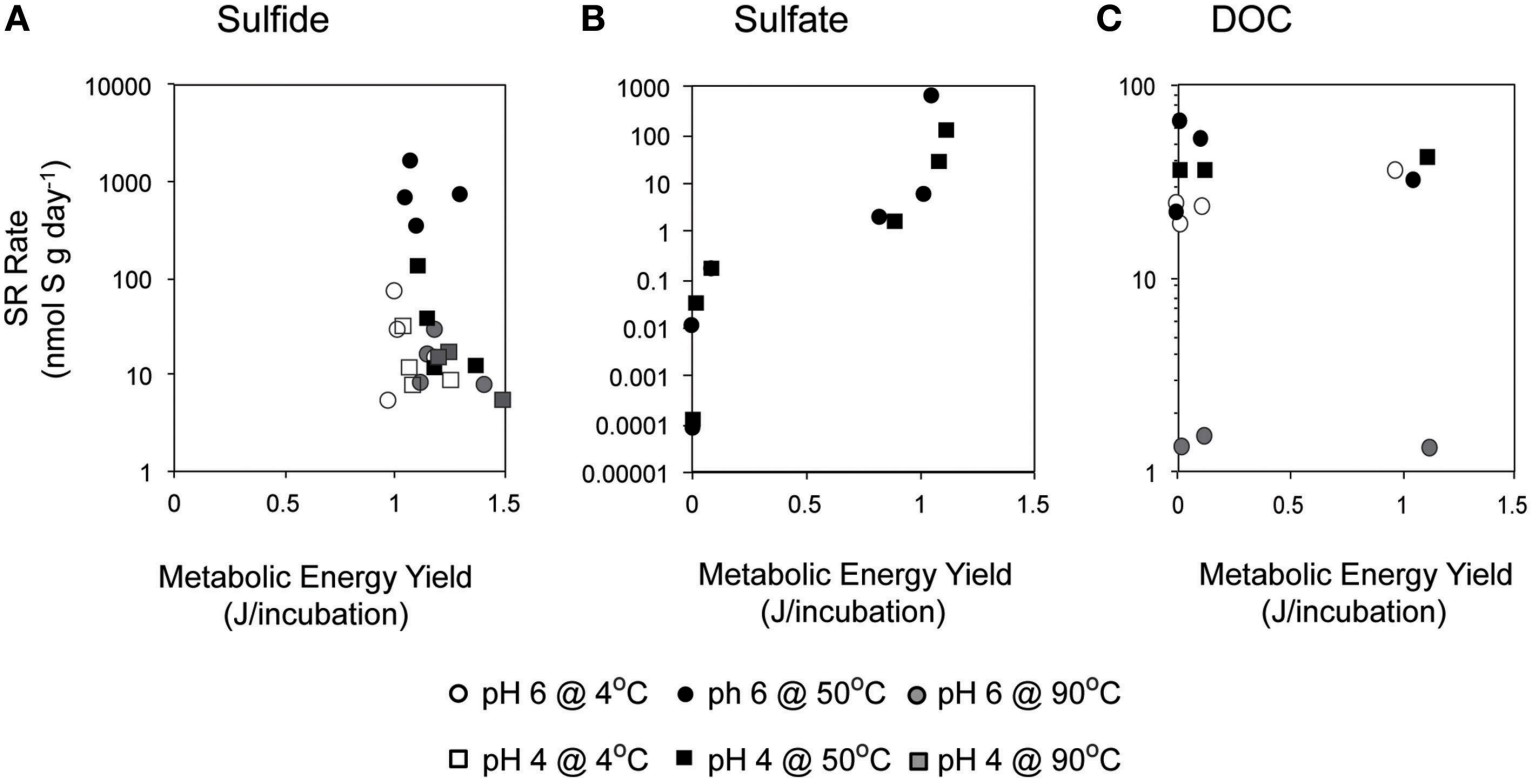
**Relationship between metabolic energy available from sulfate reduction and empirically measured rates of SR in incubations with varying concentrations of (A) sulfide, (B) sulfate, and (C) DOC are shown here at 4°C (white), 50°C (black), and 90°C (gray), and at pH 6 (circle) or pH 4 (square)**.

At 50°C and pH 4, measured SR rates increase with increasing sulfide (Figure 4B). The positive correlation of SR with sulfide concentration at pH 4 is surprising given that the favorability (Δ*G*_*r*_°) of SR decreases with increasing concentrations of sulfide. However, there are no significant correlations between rates of SR and Δ*G*_*r*_ in our sulfide amended incubations (Figure 7A; Pearson's correlation, *p* > 0.2) and no obvious patterns in energy density that might explain the differences attributable to pH. Accordingly, we investigated the possibility that sulfide amendments were poising the incubations at a redox potential favorable for SR (usually confined to E_h_ values of −0.3 to 0.0 V at circumneutral pH and slightly more positive under more acidic conditions; Lovley and Goodwin, 1988; Church et al., 2007). While the addition of sulfide at pH 4 does lower the E_h_ of the H_2_S/${\text{SO}}_{4}^{2-}$ redox pair, this effect was pH invariant (Figure S2) and thus we posit that E_h_ is unlikely to be the parameter controlling increasing SR rates with increasing sulfide concentrations at pH 4 and 50°C.

**Figure 8:**
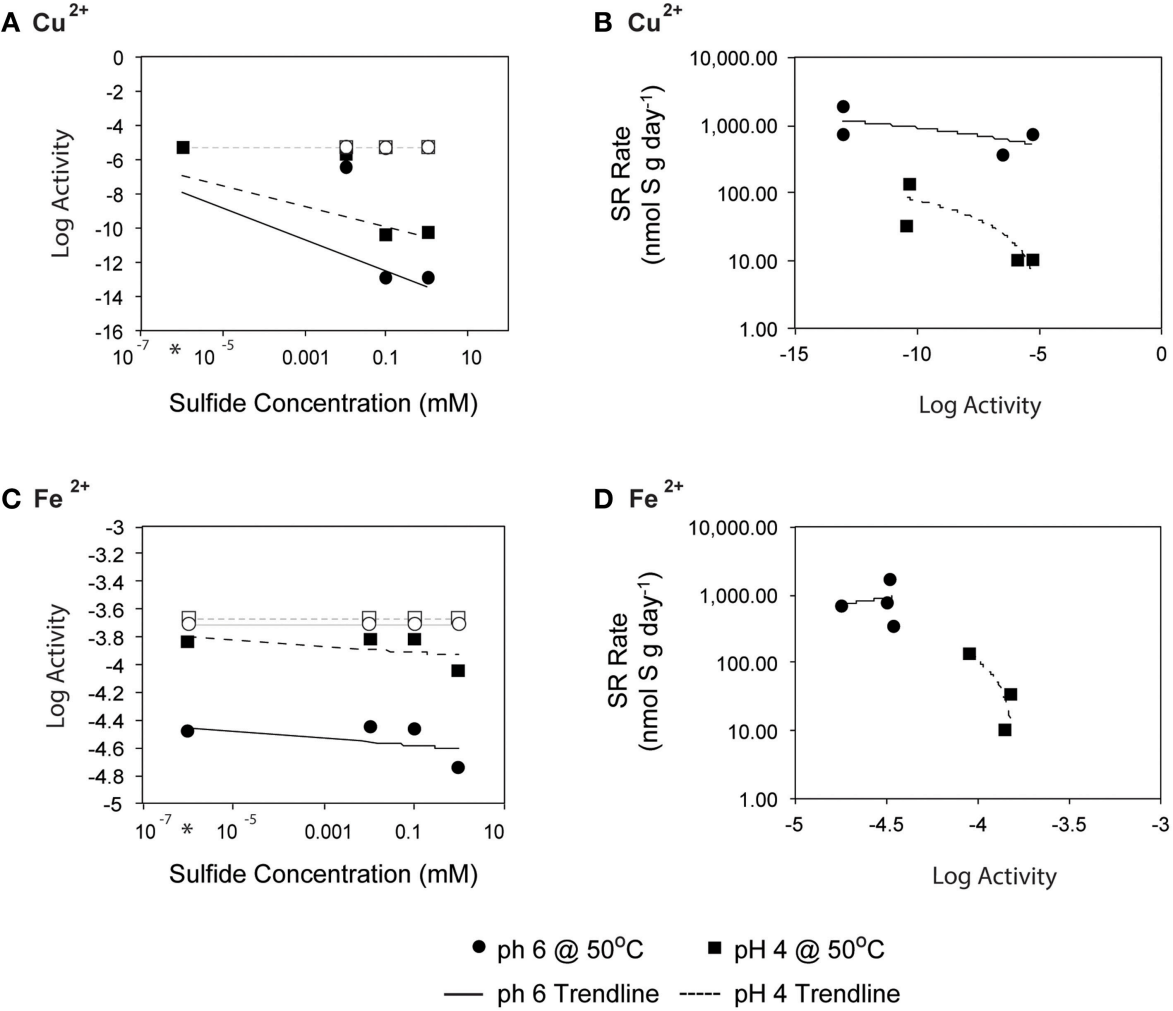
**Initial sulfide concentration and the log activities of (A) Cu^**2+**^ and (C) Fe^**2+**^ at 50°C (as calculated by EQ3/6) and pH of 4 (○) or 6 (■) are related. Linear regression lines are shown for pH 4 (- - -) and pH 6 (-)**. Average rates of SR at 50°C (from 0, 0.01, 0.1, and 1 mM H_2_S additions experiments) are plotted with respect to log activities of **(B)** Cu^2+^ and **(D)** Fe^2+^. Note that activity is a measure of the effective concentration of a species within a fluid.

Fluid pH, however, also affects mineral solubility, and at lower pH the toxicity of metals and organic acids are often elevated as minerals are solubilized and organic acids become protonated (Ghose and Wiken, 1955; Oleszkiewicz et al., 1989; Giller et al., 1998; Gadd and Griffiths, 2013). For example, at low pH, the carboxyl groups of simple organic acids are primarily undissociated, allowing them to pass across the cell membrane where they can uncouple electrochemical gradients (Ghose and Wiken, 1955). Of particular concern in hydrothermal environments is the dissolution of metal sulfides, which is enhanced at lower pH and depends on sulfide concentrations. To evaluate the potential for metal toxicity in our incubations, we calculated *in situ* activities of Fe, Zn, and Cu (assuming *in situ* end-member concentrations; Butterfield et al., 1994), which are known to be toxic when dissolved and in sufficient concentration. The activities of Fe, Zn, and Cu were elevated at lower pH, and several metal sulfides and iron oxides were supersaturated under the high aqueous sulfide incubation conditions. We investigated the possibility that high sulfide amendments drew down trace metal concentration via mineral precipitation, thus explaining the increases in SR rates with increasing sulfide concentration. To account for mineral precipitation resulting from sulfide addition, we performed a single-point reaction path calculation to constrain the expected aqueous metal activities as functions of both pH and sulfide concentration. The fluid speciation models assumed initial trace metal concentrations from end-member fluid analyses (Butterfield et al., 1994) and allowed minerals to reach supersaturation resulting in aqueous activities of ~10^−5.3^ and ~10^−3.7^, Cu^2+^ and Fe^2+^ respectively. High sulfide concentrations likely led to abiotic mineral precipitation in the incubation experiments, leading to the drawdown of trace metal concentrations. Accounting for amended sulfide concentrations, *in situ* pH, and mineral precipitation (primarily pyrite, hematite and covellite), activities of Cu^2+^ and Fe^2+^were reduced to 10^−13^ and ~10^−4.5^, respectively, depending on pH (Figure 8). Precipitation of these metal sulfides also lowered the calculated fluid pH, an effect that was more pronounced for pH 6 incubations (final fluid pH ~4 after mineral precipitation) than in pH 4 incubations (final pH ~3.3). While we could not monitor pH during the radiolabeled experiments, these values are within the range expected for Grotto chimney environments, and the effect was minimal for pH 4 incubations.

Because the sulfate reducing organisms in this study are embedded within a metal sulfide matrix, the most feasible explanation for the pattern of increasing SR rates observed at pH 4 with increasing sulfide (Figure 4B) is attenuation of metal toxicity by metal-sulfide precipitation (Figure 8). In pure cultures of *Thermococcus fumicolans, Pyrococcus* strain GB-D, *Methanocaldococcus jannaschii*, sulfide additions were shown to attenuate the toxic effects of metal cations by forming metal-sulfide complexes (Edgcomb et al., 2004). In our incubation experiments, the reduction of aqueous Cu^2+^ (and to a lesser extent Fe^2+^) via mineral precipitation, particularly at high sulfide concentrations, likely attenuated metal toxicity to native sulfate reducers. The free metal activities were below the ranges that have been found to be toxic to sulfate reducing bacteria (e.g., 10^−3.1^ to 10^−4.33^ for Cu^2+^ (Booth and Mercer, 1963; Saleh et al., 1964; Temple and Le Roux, 1964; Hao et al., 1994; Sani et al., 2001; Utgikar et al., 2002, 2003; Figure 8A). These patterns are particularly pronounced for Cu^2+^ and correlation between SR rates and aqueous Cu^2+^ activities suggests that metal-sulfide precipitation and the resulting decrease in metal toxicity can account for increasing SR rates at high sulfide concentrations (Figure 8B). The effect is even more apparent at pH 4 (e.g., a 10-fold decrease in SR rates as Cu^2+^ activities increase) likely because cells are already compromised by the low pH.

### Energetic constraints of SR rates

Substrate availability and uptake mechanisms subtly influence the magnitude of SR rates in Grotto flanges. The Michaelis-Menten constants (or *K*_*m*_, defined as the sulfate concentration at which the reaction rate is at half-maximum) calculated for Grotto are higher than the range of *K*_*m*_ values calculated for SR in marine sediments (0.204–1.63 mM; Boudreau and Westrich, 1984; Kostka et al., 2002; Roychoudhury et al., 2003; Pallud and Van Cappellen, 2006), low sulfate environments (10–70 μM; Ingvorsen et al., 1981; Lovley et al., 1982), and marine sulfate reducers in pure culture (3.0–330 μM; Ingvorsen et al., 1981; Ingvorsen and Jørgensen, 1984; Sonne-hansen and Westermann, 1999). One plausible explanation for why *K*_*m*_ values for hydrothermal SR are higher than in other marine systems, is that the cellular uptake of sulfate is less efficient in hydrothermal sulfate reducers. Since all the enzymes required to catalyze sulfate into hydrogen sulfide are located in the interior of a cell (cytoplasm or associated with the inner surface of the cytoplasmic membrane), sulfate must be transported into the cells in order to be utilized. Due to the high sulfate concentration in seawater, marine SR communities often utilize low affinity transport mechanisms such as cation dependent passive transport (Cypionka and Konstanz, 1989; Kreke and Cypionka, 1992, 1994, 1995) but can switch to active transport via sulfate permeases (Piłsyk and Paszewski, 2009) under sulfate limitation (Tarpgaard et al., 2011). In comparison to marine systems, hydrothermal sulfide deposit environments have higher metal and H_2_S loads, lower relative sulfate and experience higher temperature and lower pH fluids. Higher *K*_*m*_ values at vents may be a result of insoluble metal sulfides acting as barriers to prevent the transport of sulfate (or other substrate) into the cell (Utgikar et al., 2002). Hydrothermal communities may mediate the potentially inhibitory effects of high temperature, low pH and sulfate limitation by increasing the number or type of sulfate permeases per cell. Our data suggests that sulfate reducers have a higher affinity for sulfate at pH 4 than at pH 6 and may utilize different mechanisms (like increasing the number or type of sulfate permeases utilized) to overcome pH-induced constraints to maintain constant rates of SR.

It is also important to note that Michaelis-Menten kinetic models, and calculated values of *K*_*m*_, are based on idealized growth conditions, including optimized physiological parameters, single limiting substrates (e.g., sulfate), the absence of inhibitory mechanisms, and no community members of competition in the community structure. For example, our values of *K*_*m*_ assume that sulfate is the sole limiting substrate, however calculated energy densities (Figure 5Bv) indicate this is not always, or even usually, the case in this system. Interestingly, the measured rates, at 14 mM sulfate, are comparable in magnitude to hydrothermally influenced sediments from Guaymas basin (high sulfate; Jørgensen et al., 1990; Elsgaard et al., 1994a) and Lake Tanganyika (low sulfate; Elsgaard et al., 1994b), which have strikingly different sulfate concentrations, suggesting that sulfate concentration alone may not govern the overall kinetics of SR. Considering this, it is more likely that energy availability in the context of substrate availability is constraining the magnitude of rates and complicating the Michaelis-Menten kinetics pattern we might expect.

Substrate limitation is often concomitant with energy-limited conditions. Minimal energy requirements for autotrophic SR in marine sediments are inferred to be −19.1 kJ/mol ${\text{SO}}_{4}^{2-}$ (Hoehler et al., 2001) and it has been suggested that these minimal requirements are always easily met for heterotrophic metabolisms (Lever et al., 2015). Regardless, our values of Gibbs free energy yields, normalized per mole of sulfate (ca. −100 to −150 kJ/mol ${\text{SO}}_{4}^{2-}$), greatly exceed the minimum energy requirements for autotrophic sulfate reduction. Thus, it is unlikely that metabolic energy availability was limiting in most conditions in our experiments. Nonetheless, SR rates are positively correlated with metabolic energy availability (Figure 7B; Kendal rank correlation *p* < 0.005), suggesting that energy availability still plays a role in microbial metabolic rates, even if it is not limiting microbial growth. Furthermore, the energy available for SR across most conditions within these incubations could support ~10^5^–10^6^ cells•g matrix^−1^ [assuming that 10% of that energy supports growth, cellular synthesis requires 1434 J•g cell in anoxic environments (McCollom and Amend, 2005), the abundance of SR bacteria and archaea *in situ* is 10^3^–10^4^ cells•g matrix^−1^ (Frank et al., 2013), and cells have 1 *dsrA* gene copy (Klein et al., 2001; Kondo et al., 2004)]. Even at the lowest values of sulfate, 10 nM, the energy within the system could support ~100 cells/g mineral.

Interestingly, high rates of SR were observed even when metabolic energy yield was assumed to be low, based on the exogenous DOC concentration provided in each batch reaction (Figure 7C). This suggests that we underestimated the energy yield provided in each batch reaction and did not take into consideration potential sources of endogenous carbon within the mineral inoculum. Heterotrophic SR utilizing a wide variety of metabolic products has been shown to be thermodynamically favorable in hydrothermal environments based on concentrations in diffuse fluid (Rogers and Amend, 2006). Mineral associated products of microbial respiration or fermentation and lysed cells are likely other sources of endogenous organic carbon. Perhaps more important is the contribution of remnants from associated macrofauna as the surface of the Grotto flange was heavily colonized by tubeworms. Tube worm growth provides structural scaffolding for the concentration and precipitation of minerals during the initial stages of hydrothermal chimney and flange formation (Kristall et al., 2006) and fossilized tube structures have been observed within mineralogical samples of Grotto deposits (Tivey et al., 1999). Reasonably, dead macrofauna—heterogeneously distributed within the walls of a hydrothermal deposit—may provide localized and concentrated sources of carbon for microbial utilization and sulfate reduction.

Currently, our assumptions of the amount of carbon available within the mineral matrix of hydrothermal vents is based solely on DOC measurements from end-member and diffuse flow (Lang et al., 2006, 2010). However, these fluid measurements may only place lower bounds on carbon availability in hydrothermal systems. Unfortunately, concentrations of mineral associated carbon (or total organic carbon) have not been evaluated, due to technical complications, in hydrothermal chimneys. The only measurements of the total organic carbon (TOC) in similar mineral hosted systems across the Juan de Fuca Ridge are in sediments and values (wt%) vary between 0.06 and 0.83% and hydrolysable amino acids account for up to 3.3% of this organic carbon (Andersson et al., 2000). If we assume a maximum density of 10^8^ cells•g matrix^−1^ (Schrenk et al., 2003), and the average carbon content of 12.4 ± 6.3 fg C per oceanic bacterial cell (Fukuda et al., 1998) that's roughly 12.4 μg C•g matrix^−1^ (0.0012% by weight) that could be respired, and could support 0.0341 J•g matrix^−1^ of energy for sulfate reduction. Energy from the recycling of bacterial biomass alone would be enough to support 10^5^ cells•g matrix^−1^, and these calculations do not even include potential carbon estimations from eukaryotic input. Similarly, recent studies suggest that the degradation of subsurface microbial biomass may be an important source of organic matter in unsedimented hydrothermal systems and expands the types of heterotrophic metabolic strategies thought possible in these environments (Reeves et al., 2014). These data suggest that hydrothermal chimneys may contain a substantially greater amount of biologically available endogenous carbon to support heterotrophic metabolisms than has previously been considered by bioenergetic models. DOC and TOC measurements at vents are relatively sparse and a more thorough documentation of these values across vent fields would be incredibly valuable to re-evaluate the potential energy yields of heterotrophic metabolisms in hydrothermal systems.

We cannot overlook the possibility of substrate competition among *in situ* microorganisms utilizing alternate metabolisms. However, because these experiments were incubated in the absence of nitrate and hydrogen, potential competition with denitrifiers or any autotrophic metabolisms can be excluded in our reactions. Furthermore, data from numerous studies suggest that under these experimental conditions heterotrophic sulfate reducers would outcompete methanogenic bacteria (Oremland and Taylor, 1977; Oremland and Polcin, 1982; Lovley and Klug, 1986; Weijma et al., 2000). While many organisms capable of iron reduction have been isolated from vents (Takai et al., 2000; Kashefi et al., 2002; Hirayama et al., 2007; Sokolova et al., 2007; Slobodkina et al., 2009), the mineralogy of Grotto—reduced iron minerals (Tivey and Delaney, 1986; Delaney et al., 1992)- suggests limited influence from iron reducers under these experimental conditions.

## Concluding remarks

Multivariate experiments that couple empirically derived data and bioenergetic modeling can significantly advance our understanding of SR within complex hydrothermal systems by placing constraints on the factor(s) most likely governing SR activity at *in situ* conditions. Moving toward a better understanding of SR—and associated processes—in the natural world requires that processes are studied at conditions relevant to those found *in situ*. Indeed, the factors governing SR *in situ* are complex, sufficiently so as to make it difficult to establish causality with a high degree of certainty. That said, this work underscores the relevance of interactions among physico-chemical conditions, water-rock reactions, microbial physiology, and metabolic energetics, and their combined effect on microbial activity. These findings suggest that the variability in sulfate reduction rates reflect the response of the active microbial consortia to environmental constraints on *in situ* microbial physiology, toxicity, and the type and extent of energy limitation. These results further emphasize that the effect of *in situ* metabolic reaction energetics is minimal under energy-rich conditions (Jin and Bethke, 2007), but likely play an important role under energy-limiting conditions and during competition. The data presented here highlight the significance of sulfate reduction in hydrothermal chimneys and provide a framework for continued studies of sulfur cycling along mid-ocean ridge systems.

It is important to recognize that geochemical models of metabolic energy availability, even when considering *in situ* fluid chemistry, cannot explain microbial activity, like SR rates, in all systems. Ideally future studies would couple fine-scale *in situ* redox measurements and microbial sampling with bioenergetic modeling and direct measurements of metabolic rates in order to more explicitly constrain microbial niches across steep redox gradients. Integrating analyses of the microbial community composition and structure, metagenomic reconstructions of energy metabolisms, and measured rates of metabolic activities of hydrothermal chimney microbes can provide specific evidence to test energetic hypothesizes. Only by concurrently assessing these biological and geochemical data can we begin to understand the relationship between vent geochemistry and the prolific and diverse communities observed therein, as well as their impact on global biogeochemical cycles of sulfur and carbon.

### Conflict of interest statement

The authors declare that the research was conducted in the absence of any commercial or financial relationships that could be construed as a potential conflict of interest.
