# Supplementary material for: Key Factors Influencing Rates of Heterotrophic Sulfate Reduction in Active Seafloor Hydrothermal Massive Sulfide Deposits
Source: Front Microbiol. 2015 Dec 22;6:1449. doi: 10.3389/fmicb.2015.01449 (PMC4686611; doi:10.3389/fmicb.2015.01449)
Supplement: Supplementary file 1 [file DataSheet1.docx]

**Supplementary Material**

**Figure S1: Environmental scanning electron microscope (SEM) images of crushed Grotto flange**. Microbes (confirmed with EDAX scan of consisting primarily of carbon) are highlighted in green.

**Figure S2:** E_h_ of the H_2_S/SO_4_^2-^ redox pair was calculated using EQ3 given the chemical speciation of incubation fluid. E_h_ values at 4°C (white), 50°C (black) and 90°C (gray) at pH 6 (◼) and pH 4 (🞆) are shown here.

**Table S1: Pearson’s correlation p values**

| **Gradient:** | **Sulfide: 0-1 mM** | | **Sulfate: 10 nM – 14 mM** | | **DOC: 0 - 50 μM** | |
| --- | --- | --- | --- | --- | --- | --- |
| **Temperature** | pH 6 | pH 4 | pH 6 | pH 4 | pH 6 | pH 4 |
| **4°C** | p=0.057 | p<0.0001 | p=0.1260 | - | - | - |
| **50°C** | p=0.5156 | p<0.0001 | p=0.9659 | p=0.5038 | p<0.0001 | p<0.0001 |
| **90°C** | p=0.5306 | p=0.4340 | p=0.5654 | - | - | - |
| **All data** | p=0.0004 | | p=0.5301 | | p<0.0001 | |
