# Supplementary figures and images for: Key Factors Influencing Rates of Heterotrophic Sulfate Reduction in Active Seafloor Hydrothermal Massive Sulfide Deposits
Source: Front Microbiol. 2015 Dec 22;6:1449. doi: 10.3389/fmicb.2015.01449 (PMC4686611; doi:10.3389/fmicb.2015.01449)

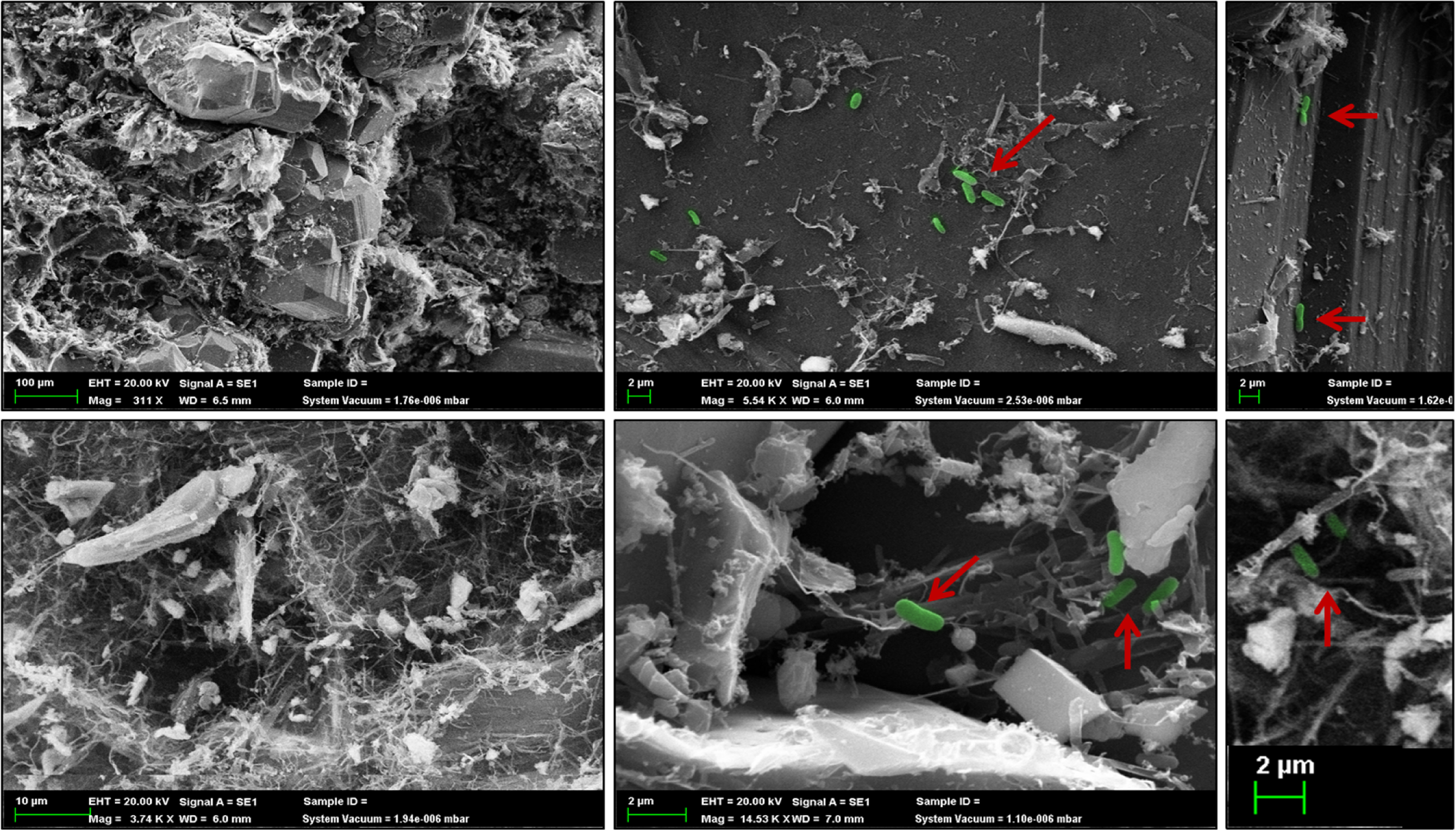

Supplement: Supplementary file 2 [file Image1.TIF]

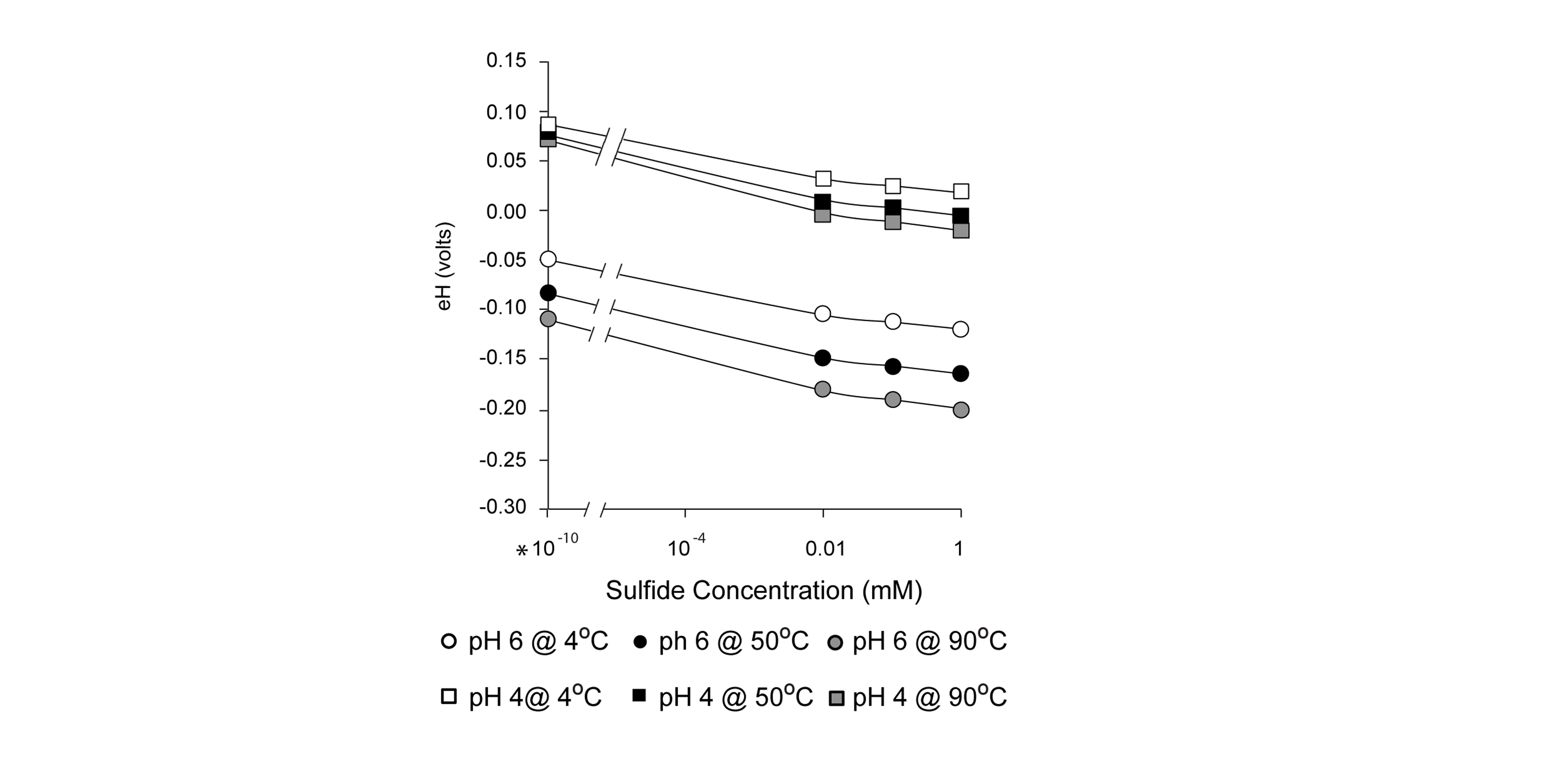

Supplement: Supplementary file 3 [file Image2.TIF]
